# Supplementary material for: Boost event-driven tactile learning with location spiking neurons
Source: Front Neurosci. 2023 Apr 21;17:1127537. doi: 10.3389/fnins.2023.1127537 (PMC10160479; doi:10.3389/fnins.2023.1127537)
Supplement: Supplementary file 1 [file Presentation_1.pdf]

frontiers

# Supplementary Material

## 1 DATASETS

This section introduces the details of the datasets for the Hybrid_SRM_FC and the datasets for the Hybrid_LIF_GNN.

### 1.1 Datasets for the Hybrid_SRM_FC

Objects-v1: There are 36 object classes in this dataset, including 26 objects directly from the YCB dataset Calli et al. (2015) and 10 deformable YCB objects. To collect each event-driven tactile data sequence, the gripper grasped an object, lifted it off the table by 20 cm, and placed it back onto the table. Each data sequence is 6.5 seconds, including the time from lifting the object to releasing it. And we sample the spikes every 0.02 seconds like Taunyazoz et al. (2020), which results in the total time length of this dataset being 325. For each object class, 25 event sequences are collected, yielding a total of 900 event sequences. Each gripper has two event-driven tactile sensors. And we use the event data from these two tactile sensors (78 taxels) in the experiments.

Containers-v1: There are 20 object classes in this dataset, including four containers with five different volumes. Four containers are a coffee can, a plastic soda bottle, a soymilk carton, and a metal tuna can. Five different volumes are 0%, 25%, 50%, 75%, 100% of the respective maximum amount of water (or rice for the tuna can). The gripper grasped a container and lifted it off the table by 5 cm to collect each tactile data sequence. Each data sequence is 6.5 seconds, including the time from grasping the container to lifting and holding it for a while. And we sample the spikes every 0.02 seconds like Taunyazoz et al. (2020), which results in the total time length of this dataset being 325. For each class, 40 event sequences are collected, yielding a total of 800 event sequences. Each gripper has two event-driven tactile sensors. And we use the event data from these two tactile sensors (78 taxels) in the experiments.

Slip Detection: Two objects are used in the experiments. One is stable and the other one is unstable. They are visually identical and have the same overall weight. The model is required to determine whether the held object is stable or rotational in a short time. To collect each event-driven tactile data sequence, the gripper is instructed to close upon the object, lift by 10cm off the table in 0.75 seconds and hold it for an additional 4.25 seconds. Since we are interested in rapid slip detection, we extract a 0.15s window around the start of the lift like Taunyazoz et al. (2020). And we sample the spikes every 0.001 seconds, which results in the total time length of this dataset being 150. For each object, 50 event sequences are collected, yielding a total of 100 event sequences. Each gripper has two event-driven tactile sensors. And we use the event data from these two tactile sensors (78 taxels) in the experiments.

Interested readers can find more details about the datasets from Taunyazoz et al. (2020) and the corresponding website[1].

### 1.2 Datasets for the Hybrid_LIF_GNN

Objects-v0: There are 36 object classes in this dataset, including 26 objects directly from the YCB dataset Calli et al. (2015) and 10 deformable YCB objects. The gripper grasped an object, lifted it off the table by 20 cm, and placed it back onto the table to collect each event-driven tactile data sequence. Each

[1] https://clear-nus.github.io/visuotactile/download.html

data sequence is 5 seconds, including the time from lifting the object to releasing it. And we sample the spikes every 0.02 seconds like Gu et al. (2020), which results in the total time length of this dataset being 250. For each object class, 20 event sequences are collected, yielding a total of 720 event sequences. Each gripper has two event-driven tactile sensors. And we use the event data from only one tactile sensor (39 taxels) in the experiments to fairly compare with other published results.

Containers-v0: There are 20 object classes in this dataset, including four containers with five different volumes. Four containers are a coffee can, a plastic soda bottle, a soymilk carton, and a metal tuna can. Five different volumes are 0%, 25%, 50%, 75%, 100% of the respective maximum amount of water (or rice for the tuna can). The gripper grasped a container and lifted it off the table by 5 cm to collect each tactile data sequence. Each data sequence is 6.5 seconds, including the time from grasping the container to lifting and holding it for a while. And we sample the spikes every 0.02 seconds like Gu et al. (2020), which results in the total time length of this dataset being 325. For each class, 15 event sequences are collected, yielding a total of 300 event sequences. Each gripper has two event-driven tactile sensors. And we use the event data from only one tactile sensor (39 taxels) in the experiments to fairly compare with other published results.

Interested readers can find more details about the datasets from Gu et al. (2020) and the corresponding website[2].

## 2 MEAN SPIKING RATES

We present the mean spiking rates of Hybrid_SRM_FC layers in Table S1. And Table S2 provides the mean spiking rates of Hybrid_LIF_GNN layers.

**Table S1.** Mean spiking rates of Hybrid_SRM_FC layers.

| | Objects-v1 | Containers-v1 | Slip Detection |
|---|---|---|---|
| Input layer | 0.1539 | 0.2316 | 0.0333 |
| Spiking Fully-connected layer1 with TSRM neurons | 0.2148 | 0.2461 | 0.0536 |
| Spiking Fully-connected layer2 with TSRM neurons | 0.0146 | 0.0176 | 0.1962 |
| Spiking Fully-connected layer1 with LSRM neurons | 0.2134 | 0.3576 | 0.0232 |
| Spiking Fully-connected layer2 with LSRM neurons | 0.0284 | 0.0376 | 0.0824 |

**Table S2.** Mean spiking rates of Hybrid_LIF_GNN layers

| | Objects-v0 | Containers-v0 |
|---|---|---|
| Input layer | 0.1272 | 0.0994 |
| Spatial Spiking Graph layer | 0.0271 | 0.0106 |
| Spatial Spiking Fully-connected layer1 | 0.1503 | 0.0857 |
| Spatial Spiking Fully-connected layer2 | 0.1767 | 0.1077 |
| Spatial Spiking Fully-connected layer3 | 0.0403 | 0.0580 |
| Temporal Spiking Graph layer | 0.0134 | 0.0067 |
| Temporal Spiking Fully-connected layer1 | 0.1100 | 0.2196 |
| Temporal Spiking Fully-connected layer2 | 0.1275 | 0.1489 |
| Temporal Spiking Fully-connected layer3 | 0.0213 | 0.0419 |

[2] https://clear-nus.github.io/visuotactile/download.html

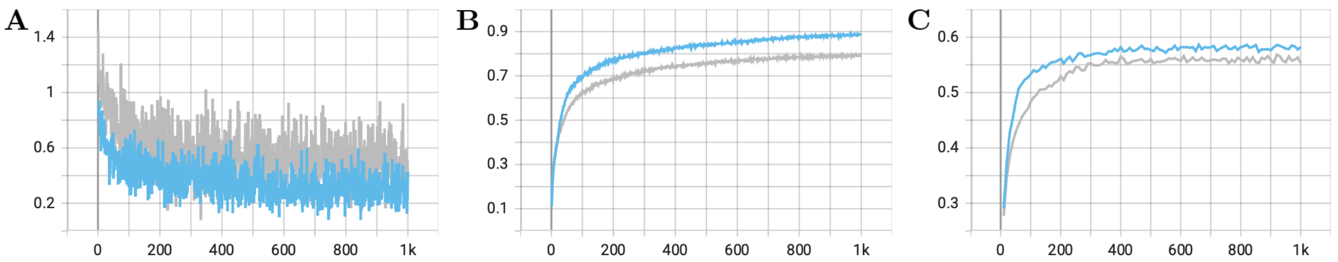


Figure S1: Training and testing profiles for the SNN with TSRM neurons (gray) and the Hybrid_SRM_FC (blue): **(A)** the training loss, **(B)** the training accuracy, **(C)** the testing accuracy.

## 3 TRAINING AND TESTING PROFILES ON EVENT-DRIVEN AUDIO LEARNING

We show the training and testing profiles of the SNN with TSRM neurons and the Hybrid_SRM_FC in Fig.S1. From these figures, we can see that our hybrid model converges faster and attains a lower loss and a higher accuracy compared to the SNN with TSRM neurons on event-driven audio learning. From this experiment, we can see that location spiking neurons can be applied to other spike-based learning applications. Moreover, the location spiking neurons can bring the benefits to the models built with conventional spiking neurons and improve their performance on the tasks.

## REFERENCES

Calli, B., Walsman, A., Singh, A., Srinivasa, S., Abbeel, P., and Dollar, A. M. (2015). Benchmarking in manipulation research: Using the yale-cmu-berkeley object and model set. *IEEE Robotics & Automation Magazine* 22, 36–52

Gu, F., Sng, W., Taunyazov, T., and Soh, H. (2020). Tactilesgnet: A spiking graph neural network for event-based tactile object recognition. In *2020 IEEE/RSJ International Conference on Intelligent Robots and Systems (IROS)* (IEEE), 9876–9882

Taunyazoz, T., Sng, W., See, H. H., Lim, B., Kuan, J., Ansari, A. F., et al. (2020). Event-driven visual-tactile sensing and learning for robots. In *Proceedings of Robotics: Science and Systems*
